# Supplementary material for: HS-GC-IMS and PCA to Characterize the Volatile Flavor Compounds in Three Sweet Cherry Cultivars and Their Wines in China
Source: Molecules. 2022 Dec 19;27(24):9056. doi: 10.3390/molecules27249056 (PMC9781699; doi:10.3390/molecules27249056)
Supplement: Supplementary file 1 [file molecules-2061022-supplementary.pdf › molecules-2061022-supplementary.pdf]

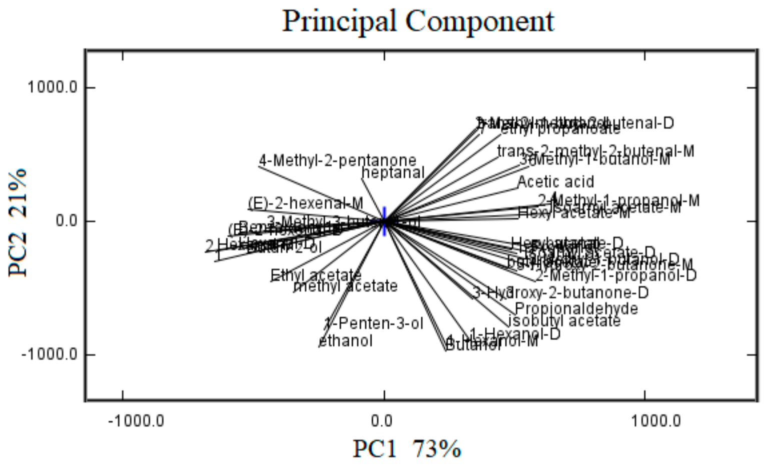

Supplementary Figure S1. The loading plot of PCA analysis in cherry fruits from three cultivars.

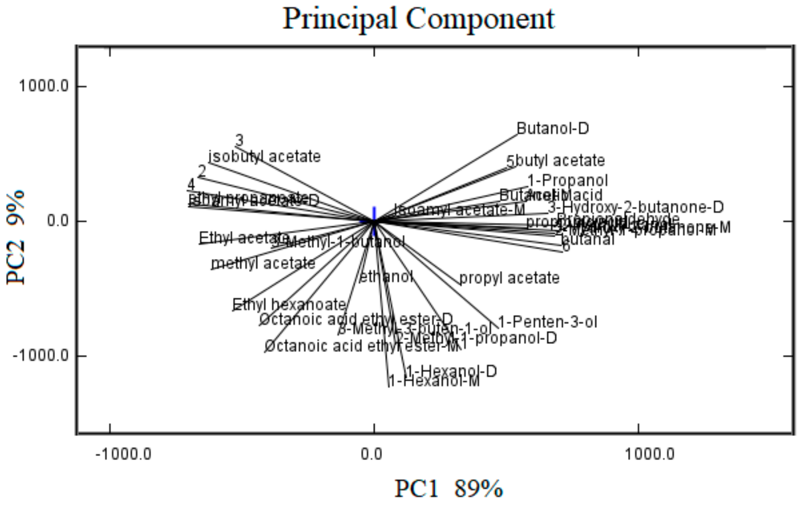

Supplementary Figure S2. The loading plot of PCA analysis in cherry wines derived from three cultivars.
